# Soil Nutrient Availability By Beneficial Bacteria of Forest Trees: From Mechanisms To Applications

**DOI:** 10.1007/s00248-026-02728-z

**Published:** 2026-02-26

**Authors:** Zhanling Wang, Qingao Wang, Yuxin Liu, Wenjun Du, Liang Hong, Dongmin Zhou, Fred O. Asiegbu, Pengfei Wu, Xiangqing Ma, Kai Wang

**Affiliations:** 1 College of Forestry, Fujian Agriculture and Forestry University, Fuzhou, 350002 China; 2 Chinese Fir Engineering Research Center of National Forestry and Grassland Administration, Fuzhou, 350002 China; 3 Department of Forest Sciences, University of Helsinki, P.O. box 27, Helsinki, FIN- 00014 Finland

**Keywords:** Beneficial bacteria, Soil nutrient, Forest management, Microbial application, Synthetic microbial community

## Abstract

As global environmental challenges intensify, enhancing forest health and soil quality has emerged as a crucial area of research. Understanding and application of beneficial bacteria in forestry industry is urgently needed as an environmentally friendly and sustainable approach. Although thousands of patents have been registered for microbial application in agriculture and forestry, the mechanisms and application of beneficial bacteria on the soil nutrient availability have not been well summarized. This review investigated the role of beneficial bacteria in tree growth, particularly their contributions to soil nutrient availability in forest trees. We summarized that beneficial bacteria significantly enhance the availability of essential elements such as nitrogen, phosphorus, potassium, and iron by promoting nutrient cycling and transformation within the soil. This process supports tree growth and improves soil quality. Additionally, beneficial bacteria facilitate plant growth by synthesizing plant hormones and inducing resistance to biotic and abiotic stresses. This review concludes by discussing practical implications of beneficial bacterial colonization and application for enhancing soil nutrient levels, along with potential future research directions. We have enriched the theoretical framework of forest-associated bacteria and provided a scientific basis that can inform forest management and ecological restoration.

## Introduction

Forest ecosystems cover approximately 31% of the Earth’s land area and play a critical role in maintaining biodiversity, regulating global carbon cycling, and buffering climate change [1]. As typical perennial systems, forests sustain long-term nutrient cycling, productivity, and ecosystem services through complex and stable plant–soil–microbe interaction networks [2]. However, climate stress, soil degradation, and human disturbances under global change increasingly threaten forest health and function. Enhancing the resilience of forest ecosystems has therefore become a key focus in current ecological and forestry research [3].

Soil microorganisms in forests, particularly beneficial bacteria closely associated with trees, act as important regulators linking soil processes to tree physiology. Rhizosphere and endophytic beneficial bacteria influence soil nutrient availability and tree nutrient uptake by participating in nitrogen fixation, phosphorus and potassium solubilization, iron chelation, and organic matter turnover [4]. These microbes also modulate plant hormone levels, enhance stress tolerance, and suppress pathogens, thereby supporting tree growth, health, and long-term adaptability [5]. A systematic review of the diversity, functional mechanisms, and ecological significance of tree-associated beneficial bacteria provides a deeper understanding of forest ecosystem processes. It also establishes a theoretical foundation for translating microbial functions into practical strategies for forest management, ecological restoration, and sustainable ecosystem stewardship.

## Classification and Diversity of Beneficial Bacteria of Forest Trees

### Classification of Beneficial Bacteria in Trees

Rhizosphere bacteria associated with forest trees inhabit the narrow soil zone influenced by roots and play key roles in regulating soil nutrient availability through nutrient mobilization, transformation, and competition with pathogens [6]. Growing evidence shows that rhizosphere community structure is strongly shaped by plant-derived metabolites, which in turn regulate microbial functions involved in biogeochemical cycling [7]. Endophytic bacteria, initially identified through early observations of microorganisms within healthy plant tissues and later formalized as “endophytes”, colonize internal plant compartments via roots, wounds, or natural openings [8]. Together with endophytic fungi and actinomycetes, they form persistent associations with host plants and are predominantly neutral, although some taxa exhibit beneficial or pathogenic effects [9]. Among these groups, beneficial bacteria represent a functionally important component that enhances tree nutrition by supporting nitrogen fixation, iron chelation, phosphorus and potassium solubilization, and by increasing host tolerance to abiotic stresses [10, 11].

From a taxonomic perspective, prokaryotes were classified into forty-two phyla by the International Committee on Systematics of Prokaryotes in 2021, while bacterial systematics remains dynamic and reflects extensive functional diversity in soil nutrient cycling [12]. The origins of beneficial rhizosphere and endophytic bacteria in forest trees are closely linked to their ecological niches and transmission pathways. Many endophytes originate from soil bacterial pools and enter plant tissues through mechanical damage or enzymatic penetration, subsequently adopting a lifestyle dependent on host-derived resources [13, 14]. Notably, some bacteria persist across plant generations through vertical or horizontal transmission, including seed-mediated inheritance under specific conditions [15]. In contrast, rhizosphere bacteria are mainly recruited from surrounding soil communities but may also derive from air, water, animals, or neighboring plants, highlighting the rhizosphere as a dynamic interface shaped by host-driven nutrient demands and a key target for microbiome-based nutrient management in forest ecosystems [16].

### Beneficial Bacterial Diversity in Rhizosphere and Endophyte of Forest Trees

Rhizosphere and endophytic bacterial communities associated with forest trees generally exhibit high levels of species diversity. This diversity provides an essential ecological basis for beneficial bacteria to participate in soil nutrient transformation and regulation. Microbial communities with high diversity tend to maintain functional stability in complex and dynamic forest soil environments. Such stability ensures the continuous operation of nutrient transformation processes across different host backgrounds and environmental conditions [17]. Studies have shown that the rhizosphere and endophytic bacteriomes of Chinese fir (*Cunninghamia lanceolata*) maintain stable diversity across different developmental stages. In contrast, their metabolic activity and functional potential vary markedly. These patterns indicate that microbial communities can adjust their functional profiles in response to host physiological status and soil nutrient availability [18]. Plant growth promoting bacterial genera such as *Burkholderia*, *Bacillus*, and *Paraburkholderia* are widely distributed in the rhizosphere of forest trees (Table 1). These taxa enhance nutrient availability through biological nitrogen fixation, phosphorus and potassium solubilization, and the production of plant growth regulating compounds [19].

**Table 1:** Representative rhizosphere and endophytic bacteria involved in soil nutrient mobilization

Genus	Species	Function	References
Bacillus	*B. subtilis*	Nitrogen fixation; enhancement of urease and dehydrogenase activities; increased soil available nitrogen	(Ng et al. 2022)
	*B. amyloliquefaciens*	Phosphate and potassium solubilization; enhanced availability of micronutrients; production of siderophores facilitating Fe uptake; improvement of soil microbial community structure	(Luo et al. 2022)
	*B. velezensis*	Nitrogen fixation; inorganic phosphorus solubilization; potassium solubilization; production of IAA, ammonia, and siderophores to promote nutrient uptake; increased soil phosphatase activity	(Zhang et al. 2024)
	*B. pumilus*	Phosphate solubilization; synergistic interaction with Pseudomonas putida to enhance mineral dissolution	(Kálmán et al. 2024)
	*B. firmus*	Phosphate solubilization; promotion of K and Mg release; increased availability of total and ammonium nitrogen; reduction of soil pH; improve of nutrient retention capacity	(Haroun et al. 2023)
Pseudomonas	*P. fluorescens*	Strong phosphate solubilization; IAA production; phytohormone-mediated enhancement of root nutrient uptake; increased Fe acquisition via siderophore production	(Bakki et al. 2024)
	*P. putida*	Phosphate and potassium solubilization; stimulation of root growth; enhanced nitrogen and phosphorus uptake efficiency	(Kálmán et al. 2024)
	*P. aeruginosa*	Phosphorus solubilization and increased soil phosphatase activity; significant increases in available sulfur, ammonium nitrogen, and potassium; activation of nutrient-transforming enzymes (e.g., urease and dehydrogenase); acceleration of organic carbon and nitrogen turnover; improvement of soil organic matter cycling and stable nutrient supply	(Haroun et al. 2023)
Paraburkholderia	*P. suaedae* (nov. sp.)	Nitrogen fixation and phosphate solubilization; production of siderophores and other plant growth–promoting traits; genomic (D10) evidence supporting enhanced nutrient availability	(Park et al. 2025)
	*Paraburkholderia* sp.	Nitrogen fixation; solubilization of phosphorus, potassium, and silicon minerals; production of phytohormones (e.g., IAA); synthesis of siderophores	(Rojas-Rojas et al. 2025)
Burkholderia	*B. graminis*	Phosphate solubilization; IAA production; siderophore-mediated iron mobilization	(Castanheira et al. 2016)
	*B. tropica*	Nitrogen fixation; phosphate solubilization; enhancement of nitrogen and phosphorus availability in nutrient-poor soils	(Ghosh et al. 2016)
	*Burkholderia* sp. (PSB isolates)	Increased availability of phosphorus, nitrogen, and potassium; restructuring of soil microbial communities	(Li et al. 2024)

The diversity of beneficial bacterial communities is reflected in species richness as well as in structural complexity and functional capacity. Community composition differs significantly among tree species, developmental stages, and environmental conditions. Environmental factors including soil type, pH, nutrient status, moisture, and temperature strongly influence the recruitment and establishment of beneficial bacteria [20]. Variation in the chemical composition and quantity of root exudates further modulates these processes. Within belowground compartments, bacterial diversity is typically higher in the rhizosphere than in root tissues [21]. This pattern is largely driven by root exudates. These exudates supply carbon sources and signaling molecules that create nutrient rich microhabitats for rhizosphere microorganisms and support the development of complex, functionally diverse communities [22]. In contrast, endophytic bacterial diversity is constrained by host selective filtering and tissue specificity. Although species richness is relatively lower, endophytic bacteria often display specialized functions and more intimate interactions with their hosts [19].

In addition, the diversity of beneficial bacteria is closely associated with their functional potential. High species richness and complex community structure provide functional redundancy. This redundancy supports the stable execution of key nutrient mobilization processes, including nitrogen fixation, phosphorus and potassium solubilization, and iron chelation [23]. Community level diversity also promotes interactions among bacterial taxa. These interactions include cooperation and competition that enhance nutrient cycling efficiency and improve host plant tolerance to abiotic stress [24]. Some endophytic bacteria can be vertically transmitted through seeds, which contributes to the persistence of host associated microbial functions. In contrast, rhizosphere bacteria are continuously replenished through horizontal transmission from soil, air, water, and neighboring plants. Together, these transmission pathways ensure the long term maintenance of functional microbial resources in forest ecosystems [25].

Overall, the diversity of beneficial bacteria in the rhizosphere and endosphere of forest trees reflects the ecological complexity of these communities. This diversity also provides the foundation for microbial contributions to tree growth promotion and the regulation of nutrient availability. A detailed understanding of these diversity patterns is essential for elucidating the mechanisms, by which beneficial bacteria enhance soil nutrient availability in forest systems.

## Mechanisms of Beneficial Bacteria in Enhancing Nutritional Status of Forest Trees

The growth of forest trees depends on the basic supply of soil nutrients and is also strongly influenced by rhizosphere and endophytic microorganisms. Recent studies show that beneficial bacteria enhance tree nutritional status through multiple mechanisms. These mechanisms include direct promotion of nutrient cycling and indirect effects mediated by hormone regulation, enhanced stress tolerance, and interactions with fungal communities. Such microbial mediated networks play a central role in maintaining forest ecosystem functions and improving tree productivity.

### Direct Mechanisms

**Fig. 1:**
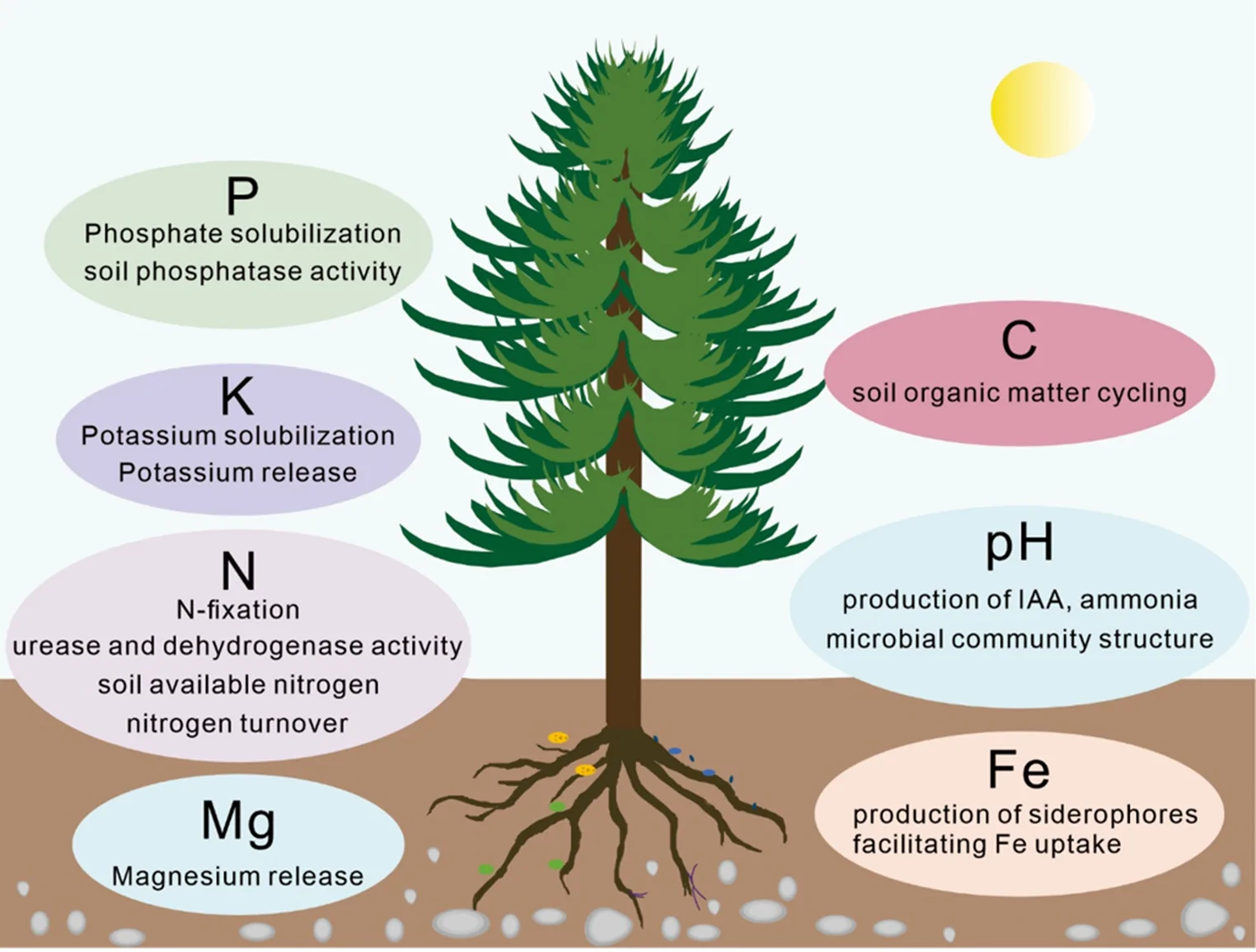
Function map of beneficial rhizosphere and endophytic bacteria in accelerating soil nutrient availability to forest trees

Beneficial bacteria directly enhance nutrient acquisition efficiency in forest trees and increase soil nutrient availability through multiple mechanisms. In the nitrogen cycle, nitrogen-fixing bacteria such as *Azotobacter* spp., *Azospirillum* spp., *Rhizobium* spp., and *Paraburkholderia* spp. convert atmospheric N₂ into ammonium that can be absorbed by roots, providing a stable nitrogen source for trees [26]. Endophytic nitrogen-fixing bacteria contribute not only to rhizosphere nitrogen fixation but also accelerate nitrogen turnover in plant tissues and soil through organic nitrogen mineralization, denitrification, and enzyme-mediated regulation (Fig. 1). They further regulate the synthesis and recycling of nitrogen-containing metabolites such as glutamate, glutamine, and theanine, offering sustained nutrient support for forests under long-term nitrogen limitation [27].

In the phosphorus cycle, most soil phosphorus exists in insoluble mineral forms. Phosphate-solubilizing bacteria release organic acids such as citric acid and oxalic acid to locally lower rhizosphere pH or synthesize acid phosphatase and phytase to convert organic phosphorus into soluble phosphate ions (PO₄³⁻). These activities also promote coupled nitrogen-phosphorus cycling in the soil, improving the overall uptake efficiency of both nutrients by trees [28]. In the potassium cycle, potassium-solubilizing bacteria release fixed potassium as K⁺ through mineral weathering, ion exchange, and biofilm-mediated interface reactions. This enhances potassium acquisition efficiency and interacts with nitrogen and phosphorus uptake to establish a multi-nutrient complementary mechanism [29].

Beneficial bacteria secrete siderophore that convert poorly soluble soil iron into bio-available forms. This transformation supports chlorophyll synthesis, maintains enzymatic activity, and facilitates signal transduction [30]. Beneficial bacteria also form biofilm and extracellular polymeric substances (EPS), which stabilize their colonization and promote soil aggregate formation. These processes reduce leaching losses of nitrogen, phosphorus, potassium, and micronutrients and create micro-scale nutrient reservoirs that provide sustained nutrient supply for trees [31].

Under nutrient-limited conditions, microbial metabolic activities optimize rhizosphere nutrient distribution and increase nutrient conversion efficiency, significantly improving root access to scarce resources. This directly supports healthy growth of forest trees and sustains ecosystem nutrient cycling. The same mechanisms also establish the microenvironment and functional foundation for indirect effects, such as hormone modulation, enhanced stress tolerance, and bacterial-fungal synergistic interactions.

### Indirect Mechanisms

**Fig. 2:**
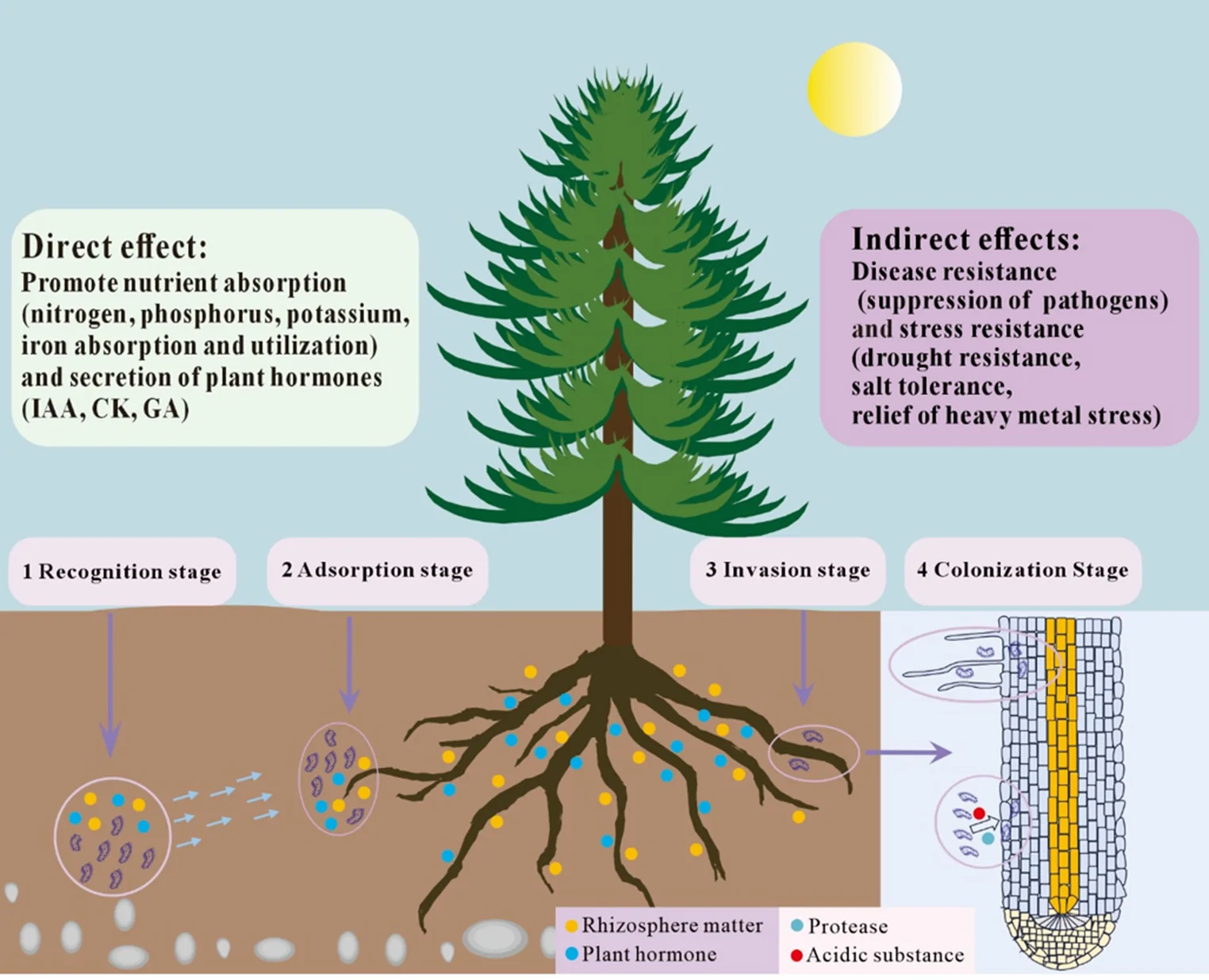
Summary of the direct and indirect effects of beneficial endopgytic bacteria to forest trees, as well the colonization steps of beneficial endophytic bacteria to plant tissue

Beneficial bacteria indirectly optimize the nutritional status and nutrient use efficiency of forest trees by regulating plant physiology, enhancing stress tolerance, and promoting cooperation with fungal communities (Fig. 2). In terms of hormonal regulation, endophytic bacteria can synthesize growth regulators such as IAA [32]. These compounds modulate root architecture and root hair distribution, thereby expanding the absorptive surface of the rhizosphere and improving nutrient uptake efficiency [33]. Certain taxa, including Variovorax spp., regulate IAA levels to maintain hormonal balance in the rhizosphere [34, 35]. This regulation supports growth stability under complex microbial assemblages. In addition, endophytic bacteria can modulate ethylene levels. This process alleviates growth inhibition under stress conditions and indirectly improves nutrient utilization efficiency [36].

With respect to stress tolerance, beneficial bacteria suppress pathogen proliferation through niche occupation, resource competition, and quorum sensing mediated regulation of antibiotic production [37]. This activity reduces nutrient losses caused by pathogenic consumption. Beneficial bacteria can also trigger induced systemic tolerance (IST) and regulate secondary metabolism [38]. These responses enhance tree tolerance to biotic stresses such as pathogens and nematodes, as well as abiotic stresses including salinity, drought, and heavy metal exposure [33]. Under saline or drought conditions, endophytic bacteria regulate ion channels and osmolyte accumulation. These processes maintain ionic homeostasis in the rhizosphere and reduce oxidative damage through enhanced antioxidant enzyme activity, thereby sustaining root function and nutrient uptake [39]. Under heavy metal stress, beneficial bacteria reduce metal bioavailability through chelator secretion, rhizosphere acidification, or regulation of plant metal transporter activity [40]. These mechanisms protect the uptake of nitrogen, phosphorus, potassium, and micronutrients.

In addition, beneficial bacteria interact with rhizosphere and endophytic fungal communities to regulate fungal community structure and function. These interactions promote the colonization and metabolic activity of mycorrhizal fungi or phosphate solubilizing fungi, which enhances mineral nutrient release and transport efficiency [41]. Microorganisms jointly construct biofilm and extracellular polymeric substances [42]. This cooperation stabilizes the rhizosphere microenvironment and forms localized nutrient reservoirs that provide a stable and adjustable nutrient supply to trees. Such multilayered microbial cooperation supports tree growth under nutrient limited conditions. It also enhances adaptability to environmental variability and enables sustained nutrient acquisition over time.

In summary, beneficial bacteria mediate nutrient cycling through direct mechanisms and optimize plant physiology and microbial community structure through indirect pathways. Together, these processes form a multilayered and multifunctional nutrient promoting network. This network strengthens the nutritional status, productivity, and stress resilience of forest trees and provides a robust theoretical foundation and practical framework for sustainable forest management and ecological restoration.

## Colonization and Application of Beneficial Bacteria To Increase Soil Nutrient

### Rhizosphere Recruitment and Colonization Processes

Beneficial bacteria associated with forest trees establish functional interactions with their hosts through complex processes of rhizosphere recruitment and colonization (Fig. 2). Rhizosphere recruitment is initially driven by tree root exudates, including low molecular weight carbon compounds, organic acids, amino acids, and secondary metabolites. These compounds provide nutritional resources for microorganisms and function as chemotactic signals that attract specific bacterial populations toward the root system [37, 43]. The composition of root exudates is regulated by tree species, genotype, developmental stage, and environmental conditions. As a result, recruitment patterns of beneficial bacteria differ markedly among host trees. The recruitment process can also be influenced by insects, soil particles, and surrounding microbial communities. These components may function as carriers or intermediates that facilitate bacterial movement toward roots [5].

Following recruitment, bacteria undergo a series of colonization stages. During the recognition stage, bacteria perceive host derived signals, including flavonoids, plant hormones, and specific volatile compounds [44]. This perception enables bacteria to identify suitable colonization sites and activates chemotactic responses and intracellular signaling cascades [45]. During the adhesion stage, bacteria accumulate in nutrient enriched microsites on the root surface. They establish stable populations through attachment, adsorption, and biofilm formation, while extracellular polysaccharides and adhesion related proteins enhance tolerance to environmental stress [46]. In the invasion stage, a subset of endophytic bacteria penetrates root tissues by secreting cell wall degrading enzymes and proteases. These enzymes facilitate entry through epidermal cells and enable interactions with phloem or xylem tissues [47]. During the stable colonization stage, bacteria form persistent symbiotic populations in root hairs, root tips, or other plant tissues. They modulate host immune responses and regulate reactive oxygen species levels. At the same time, they continuously produce plant growth promoting compounds, including phytohormones, antioxidants, and nutrient solubilizing enzymes, which sustain mutualistic interactions [48].

The entire recruitment and colonization process is regulated by multiple factors, including host genotype, rhizosphere microbial network structure, soil physicochemical properties, and environmental stresses such as drought, salinity, or pathogen pressure [49]. These interactions display strong host specificity and dynamic adaptability [50]. Successfully colonized beneficial bacteria enhance rhizosphere nutrient mobilization and improve plant stress tolerance. They also contribute to long term forest health and productivity by stabilizing soil microbial networks, promoting multi nutrient complementarity, and supporting ecosystem level functions [51]. Collectively, these processes highlight the complexity of forest tree and beneficial bacteria interactions and provide a theoretical basis for microbe driven forest ecosystem management.

### Application Studies and Their Effects

In the practice of forestry production, the application of beneficial bacteria is increasingly becoming a focal point. These microorganisms demonstrate extraordinary potential and significant effectiveness in enhancing soil fertility and promoting plant growth. Improving soil fertility and enhancing its ability to retain moisture and nutrients are crucial for ensuring robust plant development. Beneficial bacteria play a vital role in improving soil quality through their unique physiological and biochemical functions. For instance, genera such as *Bacillus* and *Pseudomonas* can efficiently convert minerals and insoluble phosphorus and potassium elements in the soil into forms that are easily absorbed and utilized by plants [52]. This conversion significantly enhances the biological availability of soil nutrients. Additionally, root-modulating bacteria form symbiotic relationships with leguminous plants [53]. This relationship not only facilitates the biological fixation of atmospheric nitrogen, providing a valuable nitrogen source for plants, but also greatly promotes plant growth [53]. This characteristic is widely utilized for biological nitrogen fixation and soil improvement, which holds significant application value in forestry production. Furthermore, *Pseudomonas* sp. AJ15 reduces metal pollution through various mechanisms, including bio-adsorption, complexation, precipitation, and oxidation [54]. This reduction mitigates the toxicity of metals on plant growth.

In the face of the complex challenges encountered during plant growth, including biotic and abiotic stresses, it is particularly important to explore and implement efficient and safe growth-promoting strategies. Beneficial bacteria have demonstrated unique advantages in this regard. For example, *Burkholderia* species not only enhance the absorption of essential nutrients by promoting biological nitrogen fixation and phosphorus solubilization but also comprehensively promote plant growth and disease resistance through multiple pathways. These pathways include the secretion of iron carriers, the production of plant hormones, and the release of antimicrobial substances [55, 56]. Similarly, *Bacillus cereus* enhances plant growth and increases stress resistance through its ability to produce IAA, 1-aminocyclopropane-1-carboxylic acid (ACC) deaminase, and phosphate-solubilizing enzymes. Additionally, it can produce extracellular enzymes and antibiotic lipopeptides or induce systemic resistance, thereby indirectly stimulating plant growth [57]. Moreover, *Bacillus subtilis*, which possesses both biological control and plant growth-promoting functions, is widely used as a biocontrol agent and soil conditioner [58]. For instance, the domestic product “Baikang” effectively suppresses pathogens through mechanisms such as nutrient competition and niche occupation [59]. This product demonstrates excellent control effects against various plant diseases, thereby providing strong support for the sustainable development of forestry production.

Although single microbial strains demonstrate clear functions under controlled experimental conditions, their performance in complex forest environments is often unstable. As microbial ecology has shifted from studying single-strain functions to exploring community-level mechanisms, traditional single-strain inoculant approaches have revealed limitations, including poor environmental adaptability and low reproducibility in forest settings [60]. In this context, the concept of Synthetic Microbial Communities (SynComs) has emerged, providing a novel approach for precise manipulation of plant root-associated microbiomes. SynComs are constructed by selecting strains with well-defined functional traits and strong ecological adaptability and combining them according to specific design principles. They can simulate key interactions of natural microbial communities in controlled systems, enabling a strategy of multi-functional integration and community-level stability regulation [61].

**Fig. 3:**
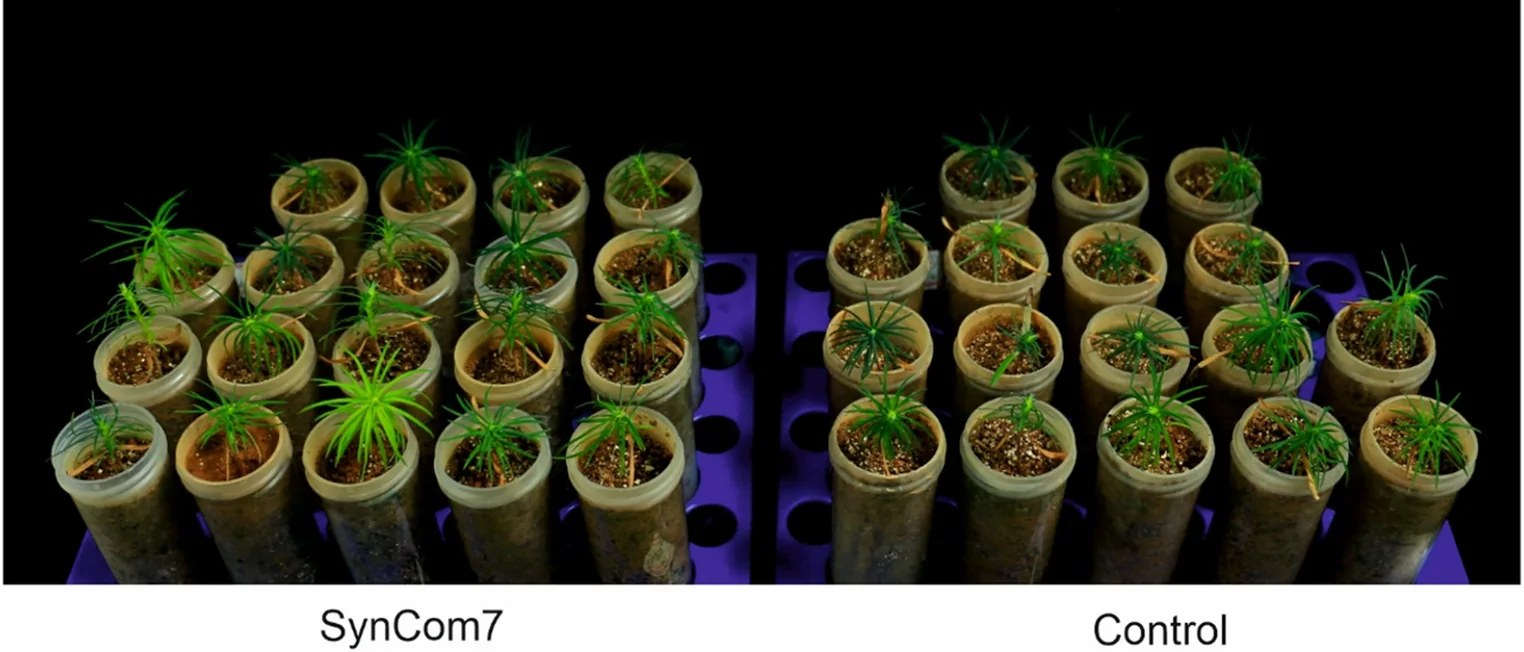
Increased growth of Chinese fir seedlings by bacterial SynCom7 (7 endophytic bacteria: 3 *Pseudomonas* spp., 2 *Bacillus* spp. and 2 *Paraburkholderia* spp.) compared to cntrol (10 mM MgCl2). Each bacteria was prepared into 2x106 cells/ml with 10 uM MgCl2. Equal volume of each bacterial solution was mixed as SynCom for soil-grown seedling treatment

To evaluate the functional performance and stability of SynComs in plant rhizosphere, researchers have developed a variety of tracking and characterization strategies. Jing et al. come up multiple strategies for tailing the SynComs, such as phenotype-based, microbiome member-based, functional gene-based, niche-based, etc [62]. Microbiota-level of gene expression during root colonization process is achievable in model plant, and the study revealed that translation and energy production processes in planta is correlated with abundance of bacteria strains [63]. In addition, the dynamics of microbial community in trees shall be inspected with similar quantitative phase and fluorescence microscopy techniques [64]. Our group has tested a SynCom (seven endophytic bacteria: 3 *Pseudomonas* spp., 2 *Bacillus* spp., 2 *Paraburkholderia* spp.) with increased growth of both above- and below-ground parts of Chinese fir seedlings, showing the potential of endophytic bacterial SynCom in improving the production of Chinese fir forest in low-phosphate soil (Fig. 3). Yet, further study is needed to seek the beneficial mechanisms, such as increased soil nutrient availability, for the SynCom to utmost application in forestry management and production.

### Limitations and Methodological Challenges

Although beneficial bacteria of forest trees have demonstrated substantial potential in enhancing nutrient acquisition, improving stress tolerance, and maintaining plant health, both their investigation and application remain constrained by multiple ecological and methodological limitations. Forest ecosystems are characterized by high biological complexity and long-term stability. These features fundamentally distinguish forests from agricultural systems and model plant frameworks, and they impose significant barriers to the consistent performance of microbial applications.

Forest soils harbor highly diverse and structurally stable microbial communities that are shaped by long-term plant–soil feedbacks [65]. This complex community background imposes strong ecological filtering on introduced or experimentally enriched beneficial bacteria. As a result, such bacteria often exhibit limited establishment, reduced functional expression, or rapid displacement by resident microbiota [66]. Consequently, beneficial effects observed under controlled conditions or short-term experiments are frequently difficult to reproduce in forest environments. This limitation reduces the reliability and predictability of current application strategies.

Host specificity further constrains the general applicability of microbial interventions in forestry. Differences among tree species, developmental stages, and genotypes strongly influence microbial recruitment, colonization efficiency, and functional performance [67]. However, many application-oriented studies rely on simplified host systems and do not adequately account for genotype-dependent responses. This limitation restricts the reproducibility of results across forest stands. The long life cycle of woody plants and their pronounced genetic heterogeneity further complicate experimental replication and mechanistic analysis [51].

From a methodological perspective, the widespread adoption of high-throughput sequencing and multi-omics approaches has greatly advanced descriptive knowledge of forest-associated bacterial communities and their potential functions. However, causal inference remains challenging [68]. Most studies are based primarily on correlations between microbial taxa and plant traits or soil nutrient status. Experimental validation using synthetic communities, gnotobiotic systems, or targeted functional manipulation remains limited. This limitation is partly due to the technical difficulty of establishing sterile or semi-sterile conditions in woody plants. As a consequence, community-level patterns are often difficult to translate into mechanistic understanding [69].

Mismatches in temporal and spatial scales represent another central challenge in this field. Forest nutrient cycling and tree growth processes operate over long time horizons [70]. In contrast, most functional evaluations of beneficial bacteria are conducted using short-term seedling or pot experiments. Short-term physiological indicators or biomass responses therefore fail to accurately reflect the long-term contributions of beneficial bacteria to soil nutrient pool maintenance, productivity stability at the stand level, and ecosystem functioning [23].

In addition, forest microbial communities generally exhibit a high degree of functional redundancy. Identical or similar nutrient transformation functions are often shared among multiple bacterial taxa [65]. As a result, the effects of single-strain inoculation are easily diluted or masked at the community level. Current studies rarely integrate microbial network structure, functional redundancy, and ecosystem stability into a unified analytical framework. This limitation constrains the identification of key functional taxa and their ecological roles [71].

At the application level, the practical deployment of beneficial bacteria in forestry remains challenged by issues related to operational feasibility and risk assessment. Technical systems for large-scale inoculum production, long-term survival and functional persistence, delivery methods, and cost control are not yet fully established [72]. In parallel, standardized frameworks for ecological risk assessment remain underdeveloped. This gap is particularly evident with respect to the potential impacts of introduced microorganisms on native microbial diversity, soil processes, and ecosystem safety [73]. Overall, these constraints indicate that, despite promising experimental evidence, the practical application of beneficial bacteria in forest systems remains limited by unresolved ecological complexity and methodological bottlenecks.

## Future Perspectives

Forest trees host highly diverse endophytic bacteria. These microorganisms play a central role in maintaining forest ecosystem functions. They promote tree growth and enhance tolerance to abiotic stresses and pathogen attacks [74]. Recent advances in strain isolation, functional characterization, and molecular identification have revealed numerous potential functional strains. However, our understanding of their ecological roles and practical application potential remains limited. Some endophytes produce growth-promoting metabolites. They also use quorum sensing to modulate microbial networks, host physiology, and pathogen suppression [75]. Environmental heterogeneity, human disturbances, and the long life-cycle of trees present major challenges for translating these findings into sustainable forestry practices [3].

Advancing our understanding of forest-associated endophytic bacteria requires a concerted focus on five interconnected research directions. First, context-dependent functional validation is essential, as the beneficial effects of endophytes are often highly contingent on environmental conditions. Developing standardized, ecologically relevant evaluation frameworks will be critical to ensure reproducible outcomes across heterogeneous forest ecosystems. Second, comprehensive exploration of microbial diversity remains a priority. Currently characterized endophytes represent only a small fraction of forest microbial diversity, and systematic discovery coupled with functional characterization of unexamined strains is likely to uncover novel mechanisms underlying growth promotion, stress resilience, and biocontrol. Third, mechanistic elucidation is needed to resolve the complex, multi-layered molecular networks that mediate tree–endophyte interactions. Integrating genomics, transcriptomics, metabolomics, and systems biology approaches will provide the mechanistic insight required for the rational design of microbial interventions. Fourth, enhancing persistence and heritability is critical, as the benefits of bioinoculants are often transient. Multi-omics-guided strategies and advanced genetic tools can support stable colonization, sustained functionality, and intergenerational transmission, thereby increasing the reliability of microbial applications in long-lived forest trees [76, 77]. Fifth, microbiome integration and community-level optimization must be carefully considered. Introducing novel strains or consortia into native microbial communities demands approaches that maintain community stability and functionality, while optimizing interspecies interactions and preserving diversity to maximize root health, nutrient acquisition, and ecosystem resilience [78] .

Overall, it is essential to shift from descriptive studies to deep research that are mechanistic, integrative, and application-oriented. Integrating molecular-level insights with ecological and evolutionary perspectives will enable forest endophyte research to deliver robust and scalable solutions for sustainable forestry under global environmental change.

## Data Availability

The data are presented within this manuscript.
